# Gut microbiota and metabolite alterations associated with reduced bone mineral density or bone metabolic indexes in postmenopausal osteoporosis

**DOI:** 10.18632/aging.103168

**Published:** 2020-05-11

**Authors:** Jianquan He, Shuangbin Xu, Bangzhou Zhang, Chuanxing Xiao, Zhangran Chen, Fuyou Si, Jifan Fu, Xiaomei Lin, Guohua Zheng, Guangchuang Yu, Jian Chen

**Affiliations:** 1 College of Rehabilitation Medicine, Fujian University of Traditional Chinese Medicine, Fuzhou 350122, China; 2 Department of Rehabilitation, Zhongshan Hospital Xiamen University, Xiamen 361004, China; 3 Department of Bioinformatics, School of Basic Medical Sciences, Southern Medical University, Guangzhou 510515, China; 4 Institute for Microbial Ecology, School of Medicine, Xiamen University, Xiamen 361102, China; 5 Department of Rehabilitation, Xinyu People's Hospital, Xinyu 338000, China; 6 College of Nursing and Health Management, Shanghai University of Medicine and Health Sciences, Shanghai 201318, China

**Keywords:** postmenopausal osteoporosis, gut microbiota, 16S rRNA gene sequencing, LC-MS metabolomics

## Abstract

Reduced bone mineral density (BMD) is associated with an altered microbiota in senile osteoporosis. However, the relationship among gut microbiota, BMD and bone metabolic indexes remains unknown in postmenopausal osteoporosis. In this study, fecal microbiota profiles for 106 postmenopausal individuals with osteopenia (n=33) or osteoporosis (n=42) or with normal BMD (n=31) were determined. An integrated 16S rRNA gene sequencing and LC-MS-based metabolomics approach was applied to explore the association of estrogen-reduced osteoporosis with the gut microbiota and fecal metabolic phenotype. Adjustments were made using several statistical models for potential confounding variables identified from the literature. The results demonstrated decreased bacterial richness and diversity in postmenopausal osteoporosis. Additionally, showed significant differences in abundance levels among phyla and genera in the gut microbial community were found. Moreover, postmenopausal osteopenia-enriched N-acetylmannosamine correlated negatively with BMD, and distinguishing metabolites were closely associated with gut bacterial variation. Both serum procollagen type I N propeptide (P1NP) and C-terminal telopeptide of type I collagen (CTX-1) correlated positively with osteopenia-enriched *Allisonella*, *Klebsiella* and *Megasphaera*. However, we did not find a significant correlation between bacterial diversity and estrogen. These observations will lead to a better understanding of the relationship between bone homeostasis and the microbiota in postmenopausal osteoporosis.

## INTRODUCTION

Postmenopausal osteoporosis (PMO) is an estrogen deficiency-induced metabolic bone disorder characterized by reduced bone mass and microarchitectural deterioration that increases the risk of bone fragility and susceptibility to fracture in postmenopausal women [1]. Approximately 10% of the world’s population and over 30% of postmenopausal women aged over 50 years suffer from osteoporosis [1–3]. Aging, estrogen deficiency, continuous calcium loss and smoking are strong independent risk factors for a high risk of PMO [1, 4]. In general, osteoporotic fracture imposes great public health, medical, and economic burdens [5–7].

The microorganisms that inhabit the gastrointestinal tract are known collectively as the gut microbiota, which consists of approximately 10 trillion bacteria [8]. Importantly, the intestinal microbiome contributes to the pathogenesis of multiple human chronic diseases, such as musculoskeletal diseases, neurological disorders, cardiovascular disease and liver diseases [9–13]. In addition, recent findings provide substantial evidence for the existence of a gut microbiota-bone axis [14–18], and the gut microbiota is a major regulator of bone mineral density (BMD) via the effects of the immune system [18, 19]. A previous study suggested that the gut microbiota regulates bone mass in mice by altering the immune status in bone and affecting osteoclast-mediated bone resorption [20]. The microbiome or its metabolites also induce bone remodeling, which is likely mediated by elevated serum IGF-1 levels [21]. Therefore, gut microbiota modulation may provide new therapeutic strategies to promote bone health.

To date, several studies have reported a close relationship between the intestinal microbiota and reduced BMD in elderly adults [22–24], and alterations in the gut microbiota may serve as biomarkers or therapeutic targets for individuals at high risk decreased BMD [23, 24]. Although such epidemiologic analyses demonstrate the underlying gut microbiota-bone axis mechanism in bone mineral loss and osteoporosis, they have mainly focused on senile osteoporosis instead of PMO. The pathogenesis between PMO and senile osteoporosis is completely different; in the latter type typically occurs after age 70, with low-turnover bone metabolism. The major consequence of reduced estrogen in PMO is the acceleration of bone resorption during menopause [25]. Hence, it is meaningful to study PMOs separately and explore gut microbiota-related fecal metabolic phenotype alterations, which would be helpful to further assess the role of the gut microbiota in the development of PMO and for understanding the pathophysiological mechanism.

**Figure 1:**
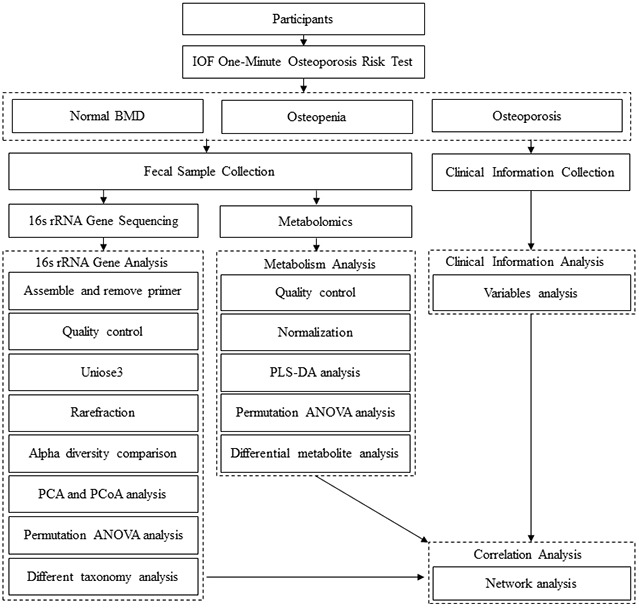
**Flow diagram of this study.** Osteoporosis: postmenopausal osteoporosis; Osteopenia: postmenopausal osteopenia.

Bone turnover markers (BTMs) are biomarkers for fracture risk that are used for the diagnosis and evaluation of the effects of therapy on PMO; the reference BTMs are serum procollagen type I N propeptide (P1NP) and serum C-terminal telopeptide of type I collagen (CTX-1), markers for bone formation and resorption, respectively [26]. Furthermore, the International Osteoporosis Foundation (IOF) recommends the use of CTX-1 and P1NP as BTMs in clinical studies on osteoporosis [27]. Nonetheless, the evidence for an association between bone metabolism and the gut microbiota in PMO remains inadequate. The aim of the present study was to investigate whether intestinal microbiota features are associated with BMD and BTMs in PMO using 16S rRNA gene sequencing and LC/MS-based metabolomics (Figure 1).

## RESULTS

### Characteristics of the participants involved in this study

In the present study, samples and clinical information for 106 individuals were analyzed. Differences in bone density measurements (T-score and BMD of the lumbar spine (L1-L4), neck of femur and total hip) and estrogen (E2) were confirmed (p<0.001, respectively), and differences in osteocalcin (OC), CTX-1 and P1NP were noted (p<0.05, respectively). No significance differences in other variables, such as age, body mass index (BMI), alcohol consumption, smoking status, the presence of common chronic diseases (e.g., hypertension, diabetes, and osteoarthritis) and medication use (e.g., angiotensin receptor blockers, metformin, and NSAIDs), among the three groups were observed (Table 1 and Supplementary Table 1).

**Table 1:** Clinical information of the participants.

**Participants, n=106**	**Normal BMD=31**	**Osteopenia=33**	**Osteoporosis=42**	**p-value**
Basic characteristics				
Age(years)	57.35±3.98	57.42±5.06	59.69±5.51	0.137
weight (kg)	60.71±6.6	58.79±7.5	57.29±5.85	0.207
BMI (kg/m^2^)	24.28±2.79	24.21±3.05	23.8±2.17	0.737
BMD				
LS Z-score ^*^	1.20±1.06	-0.49±0.62	-1.65±0.74	<0.001
LS T-score ^*^	0.06±0.93	-1.69±0.56	-3.14±0.62	<0.001
LS BMD(g/cm^2^) ^*^	1.19±0.11	0.98±0.07	0.80±0.07	<0.001
FN Z-score ^*^	1.10±0.74	-0.12±0.62	-0.83±0.76	<0.001
FN T-score ^*^	-0.01±0.69	-1.16±0.77	-2.21±0.78	<0.001
FN BMD(g/cm^2^) ^*^	0.98±0.08	0.83±0.08	0.72±0.10	<0.001
Total hip Z-score ^*^	0.98±0.77	-0.10±0.66	-0.92±0.87	<0.001
Total hip T-score ^*^	0.13±0.72	-1.00±0.67	-2.00±0.92	<0.001
Total hip BMD(g/cm^2^) ^*^	1.02±0.09	0.88±0.08	0.76±0.11	<0.001
Blood indices				
E2 (pmol/L) ^*^	45.85±29.35	31.94±13.02	24.42±7.47	<0.001
25(OH)VD3 (nmol/L)	50.86±17.7	44.35±15.38	56.28±20.46	0.126
OC (ng/ml) ^#^	19.96±7.45	26.29±10.03	24.24±13.25	0.031
CTX-1(ng/ml) ^#^	0.38±0.18	0.56±0.24	0.48±0.33	0.021
P1NP (ng/ml) ^#^	54.92±21.35	70.61±26.3	64.91±43.46	0.024
PTH (pg/ml)	45.4±21.59	45.02±16.42	47.65±26.08	0.287

Group-wise comparisons of the clinical variables. Kruskal Wallis or χ^2^ statistic was used to determine significance. The values represent mean ± S.D. or number of samples per group. Significant difference, * p<0.001 ^#^ p<0.05. BMI: body mass index. LS: lumbar spine 1-4. FN: femoral neck. BMD: bone mineral density. E2: estrogen. 25(OH)D3: serum 25-hydroxyvitamin D3. OC: osteocalcin. CTX-1: type I collagen cross-linked c-telopeptide. P1NP: procollagen type 1 n-terminal propeptide. PTH: parathyroid hormone. The complete list of sample characteristics along with pairwise comparisons is available in Supplementary Table 1.

### Intestinal bacterial diversity and enterotype in postmenopausal osteopenia and osteoporosis

**Figure 2:**
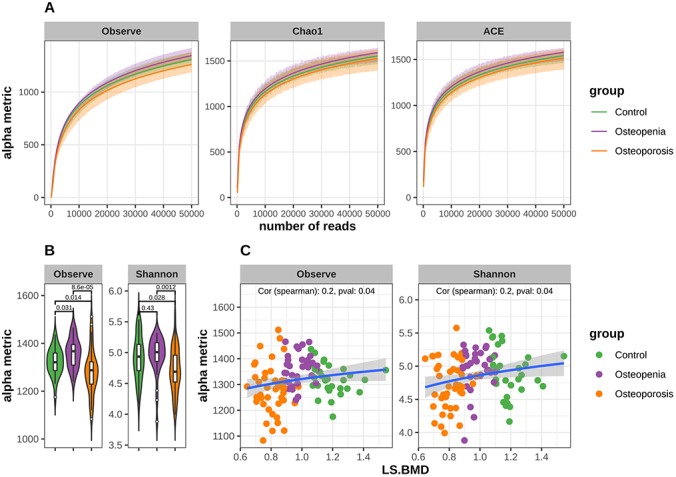
**Decreased bacterial richness and diversity in postmenopausal osteoporosis and the alpha metrics were significant associated with LS.BMD.** (**A**) Rarefaction curves for alpha richness in postmenopausal osteopenia, postmenopausal osteoporosis and control. The different facets show the different richness metric cures, the x-axis shows the number of reads, and the y-axis shows the richness metric. The shadow area shows standard deviation of each group. The curves in each group are near smooth when the number of reads is great enough with few OTUs undetected. (**B**) Comparison of α-diversity (Observe Species and Shannon) based on the OTU profile in each group. The p values are from Mann-Whitney test. (**C**) Correlation between bacterial diversity and LS.BMD. The x-axis shows the LS.BMD, and the y-axis shows the diversity values. The correlation is calculated with Spearman method.

A total of 12,463,289 high-quality reads were obtained after paired-end read merging and error correction of 16S rRNA gene sequencing data obtained from 106 stool samples, with a mean of 117,578±20,575 sequences per specimen (ranging from 74,483 to 182,563). Based on the unoise3 algorithm [28], 3323 features (OTUs) were obtained from the total high-quality reads. To check whether the sequencing data were sufficient and to characterize bacterial richness, rarefaction analysis was performed by randomly sampling 125 times with replacement and estimating the observed species, Chao1, and ACE indices calculated for these samples. The curves in each group were near saturation (Figure 2A), which suggested that the sequencing data were sufficiently robust, with very few new species undetected.

The bacterial community richness indicated by the observed species estimators was significantly lower in the PMO group than in the control or postmenopausal osteopenia group, whereas the index was significantly higher in the osteopenia group than in the control group (Figure 2B). Similarly, the community diversity estimated by the Shannon index was significantly lower in the PMO group than in the control or postmenopausal osteopenia group, and it was higher in the postmenopausal osteopenia group than in the control group and statistically significant (Figure 2B). Additionally, Spearman correlation analysis showed that LS BMD was positively associated with observed species and Shannon indices (Figure 2C), though no statistically significant correlations between E2 and the observed species or Shannon index were observed (Supplementary Figure 1). To investigate whether different enterotypes were present among the three groups, identification based on the abundance of genera was performed. The total samples clustered into three distinct enterotypes (Supplementary Figure 2A). *Prevotella_9* was the most enriched genus in enterotype 2, *Bacteroides* in enterotype 3, and *Escherichia/Shigella,* *Klebsiella* and *Phascolarctobacterium* in enterotype 1 (Supplementary Figure 2B). However, Fisher’s exact test revealed no significant differences in the percentage distribution of the different enterotypes among the three groups (Supplementary Figure 2C).

**Figure 3:**
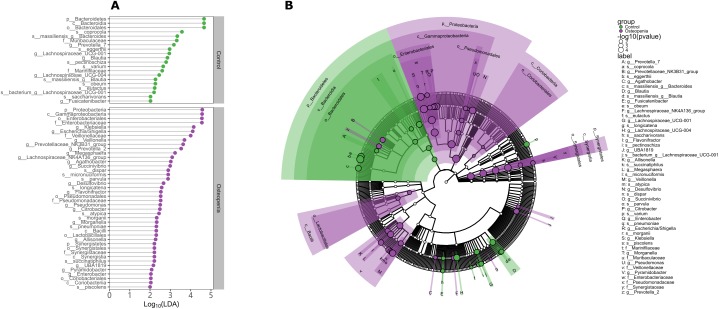
**Discriminative taxa between postmenopausal osteopenia and control.** (**A**) The point plot of LDA (Linear discriminant analysis) shows the features detected as statistically and biologically differential taxa between the different communities. (**B**) The taxonomic representation of statistically and biologically differences between postmenopausal osteopenia and control. The color of discriminative taxa represents the taxa is more abundant in the corresponding group (Control in green, postmenopausal osteopenia in purple). The size of point shows the negative logarithms (base 10) of p-value. The bigger size of point shows more significant (lower p-value).

To investigate potential differences in bacterial community structure among the groups, we further performed PCA, PCoA and PERMANOVA based on OTU abundances and found significant differences among the three groups. In detail, significant differences were observed between the postmenopausal osteopenia and control conditions, as well as between postmenopausal osteopenia and PMO; only marginally significant differences between the PMO and control conditions were found (Supplementary Figure 3 and Supplementary Table 2). In contrast, no significant association between E2 and bacterial community structure was detected (Supplementary Table 2). According to the results the Kruskal-Wallis rank sum test, Mann-Whitney test and linear discriminant analysis, there was a significantly higher abundance of Proteobacteria and Synergistetes and a significantly lower abundance of Bacteroidetes at the phylum level in the postmenopausal osteopenia group compared to the control group. At the genus level, the relative abundances of *Klebsiella*, *Morganella*, *Escherichia/Shigella*, *Enterobacter*, *Citrobacter*, *Pseudomonas*, *Succinivibrio* and *Desulfovibrio*, belonging to the Proteobacteria phylum, were significantly higher in the postmenopausal osteopenia group than in the control group (Figure 3A, 3B). Furthermore, the relative abundances of *Blautia, Fusicatenibacter*, *Lachnospiraceae_UCG-001*, *Lachnospiraceae_UCG-004* and *Prevotella_7* were significantly higher in the control group than in the postmenopausal osteopenia group (Figure 3A, 3B). At the class level, the relative abundances of Lactobacillales and Coriobacteriales were significantly higher in the PMO group than in the control group. In addition, *Parabacteroides* and *Lactobacillus* were more abundant in the osteoporosis group than in the control group (Supplementary Figure 4), and the relative abundances of *Bacteroides massiliensis*, *Lachnospira pectinoschiza*, *Bacteroides coprocola* and *Blautia* were significantly higher in the control group than in the PMO and postmenopausal osteopenia groups (Figure 3B and Supplementary Figure 4). Moreover, the relative abundances of *Megasphaera*, *Veillonella*, *Roseburia* *inulinivorans*, *Roseburia* *intestinalis*, *Klebsiella*, and *Escherichia/Shigella* were significantly higher in the postmenopausal osteopenia group than in the PMO group, but a significantly lower abundance of *Bacteroides* *eggerthii* was found in the postmenopausal osteopenia group than in the PMO group (Supplementary Figure 5).

**Figure 4:**
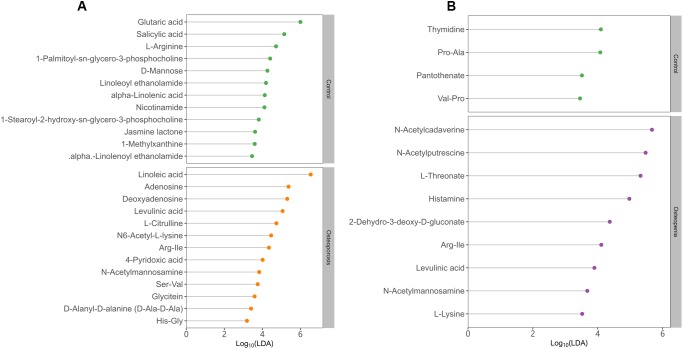
**Discriminative fecal metabolites between postmenopausal osteopenia and control.** (**A**), As well as between postmenopausal osteoporosis and control (**B**). The x-axis shows the logarithms (base 10) of LDA (Linear discriminant analysis). The y-axis shows the discriminative fecal metabolites.

### Fecal metabolism profiles in osteopenia and osteoporosis

To assess whether the profiles of fecal metabolites are associated with osteoporosis, we performed metabolic profiling of all stool samples. A significant difference in the composition of fecal metabolites was observed between the PMO group and the control group (p=0.048 and fdr=0.095 for PERMANOVA, p=0.010 and fdr=0.027 for CV-ANOVA) (Supplementary Figure 6 and Supplementary Table 3). Conversely, we observed no significant difference between the postmenopausal osteopenia group and the control group (p=0.160 and fdr=0.160 for PERMANOVA, p=0.120 and fdr=0.120 for CV-ANOVA) or between the PMO group and the postmenopausal osteopenia group (p=0.085 and fdr=0.113 for PERMANOVA, p=0.037 and fdr=0.049 for CV-ANOVA) (Supplementary Table 3).

Further stratified analysis by metabolite categories revealed that N-acetylmannosamine, deoxyadenosine, adenosine, levulinic acid, linoleic acid and Arg-Ile were significantly more abundant in the PMO group than in the control group (Figure 4B). However, glutamic acid, nicotinamide, linoleoyl ethanolamide, salicylic acid, jasmine lactone and 1-palmitoyl-sn-glycero-3-phosphocholine were significantly less abundant in the PMO group than in the control group (Figure 4A). Compared with the control group, the postmenopausal osteopenia group displayed significantly higher levels of N-acetylmannosamine, N-acetylputrescine, N-acetylcadaverine, levulinic acid, Arg-Ile and histamine but significantly lower levels of pantothenate, thymidine, Val-Pro and Pro-Ala. Interestingly, L-lysine and L-threonate were more abundant among the fecal metabolites of the postmenopausal osteopenia group than the other groups (Figure 4B). In addition, 2-hydroxy-3-methylbutyric acid, taurocholatem, N-acetylcadaverine, and histamine were more abundant in the postmenopausal osteopenia group than in the PMO group, but L-citrulline, thymidine, N6-acetyl-L-lysine and L-pipecolic acid were significantly less abundant (Supplementary Figure 7).

**Figure 5:**
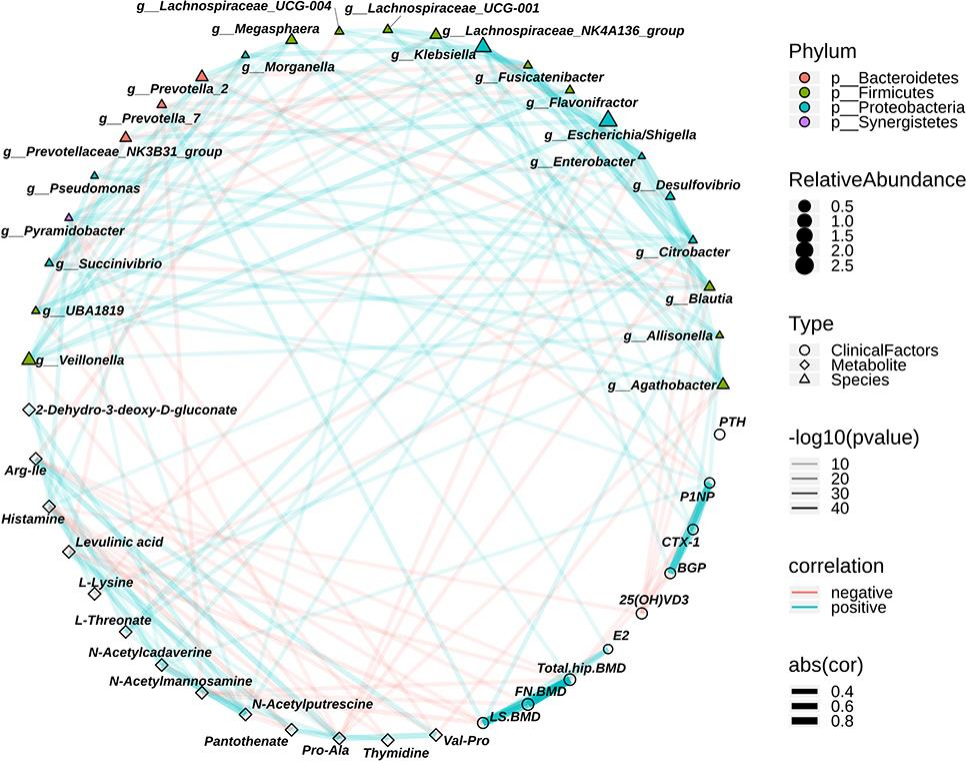
**The relationship among the discriminative genera, discriminative fecal metabolites and the clinical index associated with osteoporosis.** The colors of points show the different phyla of the genera. The size of points of genera shows the mean relative abundance. The circle points represent the clinical indexs, triangle points represent the discriminative genera, and diamond points represent the discriminative fecal metabolites. The transparency of lines represents the negative logarithms (base 10) of p-value of correlation (Spearman), the red lines represent the negative correlation and blue lines represent positive correlation, and the width of lines represents the size of correlation (Spearman).

The relationships among the different bacteria, different metabolites and clinical profilers were examined by correlation analysis (Spearman) to evaluate the relationship between the gut bacteria and fecal metabolites and between the gut bacteria and clinical profiles. We found that osteopenia-enriched N-acetylmannosamine correlated negatively with LS. BMD, FN. BMD and total hip BMD. A previous study reported that treatment with N-acetylmannosamine inhibited arthritis-mediated bone loss in mice [29]. We also found that N-acetylputrescine and N-acetylcadaverine correlated positively with N-acetylmannosamine (Figure 5 and Supplementary Table 4). Histamine, which is related to PMO, was positively associated with N-acetylcadaverine. PMO-enriched Arg-Ile correlated negatively with LS. BMD and FN. BMD (Figure 5 and Supplementary Table 4). Conversely, there was a positive association between *Prevotella*_7 enrichment in controls and BMD, including LS. BMD and total hip BMD. *Blautia* was positively associated with LS. BMD (Figure 5 and Supplementary Table 4). Interestingly, osteopenia-enriched L-threonate correlated positively with *Escherichia/Shigella*, *Enterobacter*, and *Citrobacter* (Figure 5 and Supplementary Table 4), which are from Proteobacteria and enriched in the postmenopausal osteopenia group. Postmenopausal osteopenia-enriched L-lysine correlated negatively with *Blautia* and *Fusicatenibacter* (Figure 5 and Supplementary Table 4), which were enriched in the control group. In addition, we found that osteopenia-enriched *Allisonella*, *Klebsiella* and *Megasphaera* correlated positively with P1NP and CTX-1 (Figure 5 and Supplementary Table 4). Altogether, these results indicate that the distinguishing metabolites were closely related to gut bacteria variation and that the distinguishing metabolites and intestinal bacteria were related to postmenopausal osteopenia and PMO, even though it remains to be explored whether these metabolites are directly produced by the intestinal bacteria.

## DISCUSSION

In this study, symbiotic bacteria and fecal metabolites were altered in PMO and postmenopausal osteopenia compared with control conditions. Bacterial richness and diversity were decreased in PMO. We observed that some bacteria belonging to the Proteobacteria phylum, such as *Klebsiella*, *Escherichia/Shigella*, *Enterobacter*, *Citrobacter*, *Pseudomonas*, *Succinivibrio* and *Desulfovibrio,* were enriched in postmenopausal osteopenia, and that *Parabacteroides*, *Lactobacillus* and *Bacteroides intestinalis* were more abundant in PMO. *Blautia,* *Fusicatenibacter*, *Lachnospiraceae_UCG-001*, *Lachnospiraceae_UCG-004* and *Prevotella_7* were enriched in controls. In addition, higher levels of N-acetylmannosamine, histamine, adenosine, deoxyadenosine, L-lysine and L-threonate were found in the postmenopausal osteopenia and PMO groups than in the control group. Furthermore, several distinguishing intestinal bacteria were also associated with distinguishing metabolites related to BMD.

In concert with decreases in estrogen, both bone formation and bone resorption are greatest at 7-8 years after menopause [30], correlating well with the acceleration of bone turnover observed in the osteopenia and osteoporosis groups in the present study. Moreover, osteoporosis group individuals had lower BMD than both control and osteopenia group individuals, and the cumulative loss was greater at the lumbar spine than at the hip. It has been reported that estrogen deprivation increases the permeability of the intestinal epithelium, facilitating the intrusion of intestinal pathogens, initiating immune reactions, and ultimately leading to increased osteoclastic bone resorption [31]. In this study, OC, CTX-1, and P1NP were increased in the osteopenia group compared with the control group, but they were decreased in the osteoporosis group compared with the osteopenia group (Supplementary Table 1). The bone turnover rate decreases again at approximately 10 years after menopause [30], and the average age of the individuals in the osteoporosis group was approximately 2 years older than that of the individuals in the control and osteopenia groups in our study. Hence, it is likely that the bone turnover rate was declining in some of the subjects in the osteoporosis group. We also observed both P1NP and CTX-1 to be positively associated with osteopenia-enriched *Allisonella*, *Klebsiella* and *Megasphaera*. These microbiota constituents might reflect high bone metabolic turnover in PMO.

Numerous studies in rodents have reported that alterations in the gut microbiome are associated with changes in bone mass [16, 32]. The findings of our study suggest that the α-diversity of symbiotic bacteria differed among postmenopausal osteopenia, PMO and control groups. Compared to the control condition, α-diversity was increased in postmenopausal osteopenia but decreased in osteoporosis. A study involving a few specimens showed a significant difference in α-diversity between postmenopausal osteopenia and control conditions, though reduced α-diversity was also found in PMO [22]. Another study on cohorts with reduced bone density in Ireland suggested that overall microbiota α-diversity did not correlate with BMD. We believe that these conflicting results might be due to the number of specimens and different populations of these studies. *Blautia*, *Lachnospira*, *Anaerostipes*, *Coprococcus_3*, *Fusicatenibacter*, *Lachnospiraceae_UCG-001* and *Lachnospiraceae_UCG-004*, belonging to the Lachnospiraceae family, may provide protection against colon cancer in humans by producing butyric acid and short-chain fatty acids (SCFAs) [33–35]. In our study, their abundances were decreased in the osteopenia and osteoporosis groups compared to the control group. *Blautia* comprises a group of various butyrate and acetate producers that are reported to have higher relative abundance in control subjects than in patients with type 2 diabetes mellitus [35, 36]. A beneficial anti-inflammatory association of *Blautia* has also been found in several clinical settings, including in colorectal cancer [37], cirrhosis [38], and inflammatory pouchitis following ileal pouch-anal anastomosis [39]. In the present study, we also detected a positive association of *Blautia* abundance with lumbar spine BMD, which suggests that the gut microbiota is associated with BMD. In contrast, the abundances of members of the Enterobacteriaceae and Pseudomonadaceae families, such as *Enterobacter*, *Klebsiella*, *Escherichia/Shigella*, *Citrobacter*, *Pseudomonas*, *Succinivibrio* and *Desulfovibrio*, were enriched in the osteopenia and osteoporosis groups. These bacteria belong to the Proteobacteria phylum, and recent studies have shown that mice with a disrupted microbiota exhibit reduced femur bending strength but an increased abundance of Proteobacteria. These results suggest that the abundance of Proteobacteria correlates negatively with bone mass [16], consistent with our results. Moreover, some studies have shown that the gut microbiota regulates bone metabolism through the immune system [40, 41]. The prevalence of Proteobacteria has been associated with an increased incidence of microbial dysbiosis, metabolic disease, and inflammation, all factors known to influence host physiology and the immune system [42–44]. These findings indicate that several members of Proteobacteria are associated with osteoporosis, but further studies are required to address questions on the potential detrimental impact and mechanisms of action in postmenopausal osteopenia or PMO.

Calcium absorption and metabolism are also associated with osteoporosis, as levels of calcium in the body are related to the quality and content of bone [45, 46]. Nonetheless, no distinguishing metabolites related to this metabolism were observed among the groups. We found that the abundances of adenosine and deoxyadenosine were higher among the fecal metabolites of the PMO group than the control group. Adenosine released locally mediates physiologic and pharmacologic actions via interactions with G-protein coupled receptors, and recent studies have indicated that these receptors are involved in the regulation of osteoclast differentiation and function, as well as osteoblast differentiation and bone formation [47–50]. Adenosine receptor stimulation has also been reported to improve glucocorticoid-induced osteoporosis in a rat model [47], and an experimental study in mice showed that 3’-deoxyadenosine can downregulate pro-inflammatory cytokines in an inflammation-induced osteoporosis model [51]. We also found that the abundance of N-acetylmannosamine was higher among fecal metabolites in PMO and osteopenia than in controls. A recent study showed that treatment with N-acetylmannosamine inhibited arthritis-mediated bone loss in mice. Moreover, enrichment of L-threonate and L-lysine was observed in osteopenia in our study [29]. However, laboratory studies have shown that L-lysine supplements can cause bone-building cells to be more active, with enhanced collagen production [52]. The calcium salt of L-threonate has been developed for osteoporosis treatment [53]. These findings appear to be inconsistent with our results, but much of the relevant existing literature is based on rodent studies, a small number of specimens or a specific type of osteoporosis.

Correlation analysis allowed us to identify several new bacterial genera potentially implicated in host metabolic health [54]. We found negative associations of control-enriched *Blautia* and *Fusicatenibacter* abundance with osteopenia-enriched L-lysine, whereas positive associations of *Escherichia/Shigella*, *Enterobacter* and *Citrobacter* abundances with L-threonate were observed. In addition, *Blautia* correlated positively with lumbar spine BMD, whereas levulinic acid and N-acetylmannosamine correlated negatively with lumbar spine BMD and total hip BMD. Interestingly, we found that osteopenia-enriched histamine correlated positively with *Citrobacter* and *Morganella* abundances. Previous studies have demonstrated that isolates of the two genera produce histamine [55–57]. Notably, recent research has indicated that histamine deficiency directly protects the skeleton from osteoporosis [58], suggesting a potential mechanism through which metabolites affect bone parameters via gut bacteria. It has also been reported that interleukin-33 (IL-33) elicits an inflammatory response synergistically with histamine [59] and plays an important role in regulating components of the microbiome [60]. IL-33 also represents a significant bone-protecting cytokine that may be beneficial in treating bone resorption in PMO [61]. Therefore, the relationship between IL-33 and the gut microbiome in PMO is an important research direction.

A limitation of this study was that this cross-sectional design prevented causality inference from microbiome alterations to both bone mineral loss and BTMs in PMO patients. All subjects were recruited from two communities on Xiamen Island, a small modern city in the coastal area of southern China. As the subjects were from a relatively concentrated environment, differences in geographical and climatic factors were relatively small. Nevertheless, potential dietary habits and differences may still affect the results to some extent. Hence, our findings need validation with a larger sample size in other regions. Due to the physiological interaction between organs and microbial communities, several diseases have been investigated for associations with shifts in the gut microbiome. Thus, patients with cancer, kidney disease, genetic bone disease, digestive system disease and psychiatric disease were excluded from this study. All the participants in the osteoporosis group were newly diagnosed PMO patients who had not yet received anti-osteoporotic treatment. Patients using medications such as antibiotics, probiotics, prebiotics and estrogens were also excluded, and differences in the consumption of other drugs were not significant among the three groups. Therefore, it is unlikely that medications consumed directly influenced the genomes and metabolites of the gut microbiome in these subjects. In contrast to previous studies, we applied 16S rRNA gene sequencing and quantitative fecal metabolomics, which allowed us to understand both the intestinal bacterial response and metabolites to gain additional information about host-gut microbiota metabolic interactions in response to postmenopausal osteopenia or PMO. In the future, it may be possible to develop a potential auxiliary method for the diagnosis of PMO by analysis and a proposed model for distinguishing bacteria and metabolites. Deep exploration and mechanistic studies are warranted. The deepening of knowledge about the mechanisms of intestinal bacteria shifts in PMO may provide novel targets for intervention in clinical practice.

## CONCLUSIONS

In summary, we described the disordered profiles of intestinal bacteria and fecal metabolomes in postmenopausal women with osteopenia and osteoporosis. We identified distinguishing bacteria and metabolites and discussed the relationship between them and bone parameters. These findings provide new clues regarding the link between intestinal bacteria and PMO.

## MATERIALS AND METHODS

### Study subjects

Our study included participants from Xiamen city of Fujian Province, China. The enrolled subjects were asked to complete a questionnaire regarding age, ethnicity, menstrual status, medication history, and disease history. No menstruation for at least 12 months after the last menopause was considered a postmenopausal status. Participants with cancer, kidney disease, metabolic or genetic bone disease, digestive system disease (inflammatory bowel disease, hepatic disease, constipation, previous partial or total colectomy), psychiatric disease (e.g., schizophrenia, depression, or cognitive impairment), or use of antibiotics in the past 3 months or patients using medications (e.g., estrogen, glucocorticoids, diphosphonate, teriparatide or denosumab) that might influence bone metabolism were excluded. Between 1 December 2018 and 1 February 2019, 140 postmenopausal women were screened, and 108 were found to be potentially eligible after applying IOF One-Minute Osteoporosis Risk Test [62]. In this test, those who answered YES to any of the questions of risk factor that you can change (e.g., avoiding daily foods, getting enough sunlight, little physical activity) were excluded. As a result, all eligible participants were affected by risk factors that could not be changed (e.g., age, low BMI, diabetes). The consistency of the subjects was maintained through the questionnaire. Secondary osteoporosis was detected in 2 cases, who were excluded from the analysis, resulting in a final dataset comprising 106 participants.

### Clinical measurements

Age, height (m) and weight (kg) were recorded for every participant. BMI was calculated as weight/height^2^. Daily calibrated Hologic 4500 A dual-energy X-ray absorptiometry (DXA) scanner (Lunar Expert 1313, Lunar Corp, USA) was utilized for measuring BMD (g/cm^2^) for the lumbar spine (L1-4) and total hip (femoral neck, trochanter, and intertrochanteric region). The coefficient of variation (CV), as the precision indicator, was 0.9% and 1.4% for the spine and hip BMD, respectively. BMD was recorded as the ratio of bone mineral content (g) and bone area (cm^2^), and the data are expressed as g/cm^2^. The T-score threshold was used to define three groups based on BMD. The 106 participants were divided into a control group (n=31) with a T-score of ≥-1, an osteopenia group (n=33) with a T-score between -1 and -2.5, and an osteoporosis group (n=42) with a T-score of -2.5 or less [63].

BTMs are affected by circadian variability, with peak values in the early morning and nadirs in the early afternoon and evening [64]. Therefore, we strictly collected all venous blood samples at similar time points in the morning to minimize these fluctuations. Fasting levels of BTMs, including OC, CTX-1, P1NP, parathyroid hormone (PTH), E2, and serum 25-hydroxyvitamin D3 [25(OH)VD3], were measured with an automated Roche Osteoporosis Int electrochemiluminescence system (Roche Diagnostics GmbH, Germany). The inter- and intra-assay CVs were 4.0% and 2.9% for osteocalcin, 3.5% and 2.5% for CTX-1, 2.8% and 2.3% for P1NP, 2.9% and 1.7% for PTH, 2.9% and 2.3% for E2 and 8.0% and 5.6% for 25(OH)VD3, respectively.

### Sample collection, DNA extraction, amplification, and sequencing

Fecal samples were collected in sterile plastic cups, frozen, and stored at -80°C within 1 h until further processing. Fecal microbial DNA was extracted using a QIAamp DNA Stool Mini Kit (Qiagen, Hilden, Germany). PCR amplification was carried out using an ABI 2720 Thermal Cycler (Thermo Fisher Scientific, USA). We used Multiskan™ GO spectrophotometry (Thermo Fisher Scientific, USA) to quantify bacterial genomic DNA as the template for amplification of the V3-V4 hypervariable region of the 16S rRNA gene in three replicate reactions with forward (Illumina adapter sequence 1 + 5’-CCTACGGGNBGCASCAG) and reverse (Illumina adapter sequence 2 + 5’-GGACTACNVGGGTWTCTAAT) primers. Replicate PCR products were pooled and purified with Agencourt AMPure XP magnetic beads (Beckman Coulter, USA). A TopTaq DNA Polymerase kit (Transgen, China) was used. The purity and concentration of sample DNA were assessed using a NanoDrop 2000 Spectrophotometer (Thermo Fisher Scientific, USA). Paired-end sequencing was performed by Treatgut Biotech Co., Ltd. with a HiSeq 2500 (Illumina, San Diego, CA, USA) with PE 250 bp reagents.

### Fecal metabolite extraction

Fifty milligrams of sample was placed in an EP tube, and then 1000 μL of extraction liquid containing an internal target (V methanol:V acetonitrile:V water=2:2:1, which was kept at -20°C before extraction) was added. The samples were homogenized in a bead mill for 4 min at 45 Hz and ultrasonicated for 5 min (incubated in ice water). After homogenization 3 times, the samples were incubated for 1 h at -20°C to precipitate proteins. The samples were centrifuged at 12,000 rpm for 15 min at 4°C. The supernatant (750 μL) was transferred to fresh EP tubes, and the extracts were dried in a vacuum concentrator without heating; 100 μL of extraction liquid (V acetonitrile:V water=1:1) was added for reconstitution. The samples were vortexed for 30 s, sonicated for 10 min (4°C water bath), and centrifuged for 15 min at 12,000 rpm and 4°C. The supernatant (60 μL) was transferred to a fresh 2 mL LC/MS glass vial, and 10 μL was collected from each sample and pooled as QC samples; 60 μL of supernatant was used for UHPLC-QTOF-MS analysis.

### LC-MS/MS analysis and annotation

LC-MS/MS analyses were performed using a UHPLC system (1290, Agilent Technologies) with a UPLC BEH Amide column (1.7 μm 2.1×100 mm, Waters) coupled to a TripleTOF 6600 (Q-TOF, AB Sciex) and QTOF 6550 (Agilent). The mobile phase consisted of 25 mM NH_4_OAc and 25 mM NH_4_OH in water (pH=9.75) (A) and acetonitrile (B), which was applied in an elution gradient as follows: 0 min, 95% B; 7 min, 65% B; 9 min, 40% B; 9.1 min, 95% B; and 12 min, 95% B, which was delivered at 0.5 mL/min. The injection volume was 2 μL. A TripleTOF mass spectrometer was used due to its ability to acquire MS/MS spectra on an information-dependent basis (IDA) during an LC/MS experiment. In this mode, the acquisition software (Analyst TF 1.7, AB Sciex) continuously evaluates the full-scan survey MS data as it collects and triggers the acquisition of MS/MS spectra depending on preselected criteria. In each cycle, 12 precursor ions with intensities greater than 100 were chosen for fragmentation at a collision energy (CE) of 30 V (15 MS/MS events with a product ion accumulation time of 50 msec each). ESI source conditions were set as follows: ion source gas 1 at 60 Psi, ion source gas 2 at 60 Psi, curtain gas at 35 Psi, source temperature at 650°C, and ion spray voltage floating (ISVF) at 5000 V or - 4000 V in positive or negative modes, respectively.

MS raw data files were converted to the mzXML format using ProteoWizard [65] and processed by the R package XCMS (version 3.2). The preprocessing results generated a data matrix that consisted of the retention time (RT), mass-to-charge ratio (m/z) values, and peak intensity. The R package CAMERA was used for peak annotation after XCMS data processing [66].

### Bioinformatic analyses of 16S rRNA gene sequencing

Raw paired-end reads were assembled using FLASH [28]. Primers were removed using cutadapt [67]. Chimera checking and OTU clustering were performed with the clean tags by unoise3 of usearch [28], following the pipeline [28]. In detail, all reads were demultiplexed into one file and clustered using unoise3 of usearch [28]; chimaera checking was performed using the internal function of usearch [28]. Representative sequences were generated, singletons were removed, and a final OTU table was created. Representative sequences of OTUs were aligned using the Silva database [68] for taxonomic classification with *assignTaxonomy* in the R package dada2 [69]. For downstream analysis, the feature table, taxonomy table, representative sequences, phylogenetic tree and metadata were imported and stored as a phyloseq object by the R package phyloseq [70]. The OTU table was rarefied to 50,000 reads per sample using *rarefy_even_depth* in phyloseq [70].

### Statistical analyses and visualization

Estimates of α-diversity were based on an evenly rarefied OTU abundance matrix and included observed richness for observed species, Shannon, Simpson, ACE, Chao1 indices and Pielou’s evenness (J’) using *get_alphaindex* in the in-house R package MicrobiotaProcess. The significance of differences in the measured α-diversity indices across samples were tested and visualized using nonparametric Mann-Whitney tests with *ggbox* in MicrobiotaProcess. The β-diversity, which estimates the difference in community structure between samples, of the samples was measured using the Bray-Curtis distance based on an evenly rarefied OTU abundance table. Statistical differences of the measured β-diversity metrics across groups were determined using PERMANOVA with 9999 permutations and *adonis* in R package vegan [71]. Taxon abundance was measured and plotted using *get_taxadf* and *ggbartax* in the R package MicrobiotaProcess. Taxa and metabolites with differential abundances in the groups were identified using *diff_analysis* in the R package MicrobiotaProcess, which is an algorithm for high-dimensional biomarker discovery and explanation that identifies genomic features characterizing differences between two or more biological conditions. The results of different analyses were visualized using *ggdiffclade* and *ggeffectsize* in MicrobiotaProcess. Enterotypes were identified based on the abundance of genera using kmeans. Correlations between different taxa and fecal metabolites, as well as clinical variables, were calculated by Spearman’s rank test. The results were visualized using R packages ggraph [72] and ggplot2 [73].

### Ethics statement

This study was approved by the Ethics Committee of Zhongshan Hospital, Xiamen University (No. 201808) and conducted in compliance with relevant guidelines and regulations. Written informed consent was obtained from all subjects prior to the study.

### Data availability

All associated source codes of the study can be found at the following GitHub repository: https://github.com/xiangpin/PMO_Microbiota.

## Supplementary Materials

Supplementary Figures

Supplementary Tables 1-3

Supplementary Table 4

## References

[1] Black DM, Rosen CJ. Clinical Practice. Postmenopausal Osteoporosis. N Engl J Med. 2016; 374:254–62. 10.1056/NEJMcp1513724 26789873

[2] Yu F, Xia W. The epidemiology of osteoporosis, associated fragility fractures, and management gap in China. Arch Osteoporos. 2019; 14:32. 10.1007/s11657-018-0549-y 30848398

[3] Cipriani C, Pepe J, Bertoldo F, Bianchi G, Cantatore FP, Corrado A, Di Stefano M, Frediani B, Gatti D, Giustina A, Porcelli T, Isaia G, Rossini M, et al. The epidemiology of osteoporosis in Italian postmenopausal women according to the National Bone Health Alliance (NBHA) diagnostic criteria: a multicenter cohort study. J Endocrinol Invest. 2018; 41:431–38. 10.1007/s40618-017-0761-4 28956296

[4] Bijelic R, Milicevic S, Balaban J. Risk Factors for Osteoporosis in Postmenopausal Women. Med Arh. 2017; 71:25–28. 10.5455/medarh.2017.71.25-28 28428669 PMC5364787

[5] Fischer S, Kapinos KA, Mulcahy A, Pinto L, Hayden O, Barron R. Estimating the long-term functional burden of osteoporosis-related fractures. Osteoporos Int. 2017; 28:2843–51. 10.1007/s00198-017-4110-4 28647804

[6] Darbà J, Kaskens L, Pérez-Álvarez N, Palacios S, Neyro JL, Rejas J. Disability-adjusted-life-years losses in postmenopausal women with osteoporosis: a burden of illness study. BMC Public Health. 2015; 15:324. 10.1186/s12889-015-1684-7 25880810 PMC4392468

[7] Aziziyeh R, Amin M, Habib M, Garcia Perlaza J, Szafranski K, McTavish RK, Disher T, Lüdke A, Cameron C. The burden of osteoporosis in four Latin American countries: Brazil, Mexico, Colombia, and Argentina. J Med Econ. 2019; 22:638–44. 10.1080/13696998.2019.1590843 30835577

[8] Qin J, Li R, Raes J, Arumugam M, Burgdorf KS, Manichanh C, Nielsen T, Pons N, Levenez F, Yamada T, Mende DR, Li J, Xu J, et al, and MetaHIT Consortium. A human gut microbial gene catalogue established by metagenomic sequencing. Nature. 2010; 464:59–65. 10.1038/nature08821 20203603 PMC3779803

[9] Garrett WS. The gut microbiota and colon cancer. Science. 2019; 364:1133–35. 10.1126/science.aaw2367 31221845

[10] Biver E, Berenbaum F, Valdes AM, Araujo de Carvalho I, Bindels LB, Brandi ML, Calder PC, Castronovo V, Cavalier E, Cherubini A, Cooper C, Dennison E, Franceschi C, et al. Gut microbiota and osteoarthritis management: an expert consensus of the European society for clinical and economic aspects of osteoporosis, osteoarthritis and musculoskeletal diseases (ESCEO). Ageing Res Rev. 2019; 55:100946. 10.1016/j.arr.2019.100946 31437484

[11] Castillo-Álvarez F, Marzo-Sola ME. Role of the gut microbiota in the development of various neurological diseases. Neurologia. 2019. [Epub ahead of print]. 10.1016/j.nrl.2019.03.017 35779869

[12] Jia Q, Xie Y, Lu C, Zhang A, Lu Y, Lv S, Zhang J. Endocrine organs of cardiovascular diseases: gut microbiota. J Cell Mol Med. 2019; 23:2314–23. 10.1111/jcmm.14164 30688023 PMC6433674

[13] Hrncir T, Hrncirova L, Kverka M, Tlaskalova-Hogenova H. The role of gut microbiota in intestinal and liver diseases. Lab Anim. 2019; 53:271–80. 10.1177/0023677218818605 30580671

[14] Villa CR, Ward WE, Comelli EM. Gut microbiota-bone axis. Crit Rev Food Sci Nutr. 2017; 57:1664–72. 10.1080/10408398.2015.1010034 26462599

[15] Poinsot P, Schwarzer M, Peretti N, Leulier F. The emerging connections between IGF1, the intestinal microbiome, *Lactobacillus* strains and bone growth. J Mol Endocrinol. 2018; 61:T103–13. 10.1530/JME-17-0292 29789323

[16] Guss JD, Horsfield MW, Fontenele FF, Sandoval TN, Luna M, Apoorva F, Lima SF, Bicalho RC, Singh A, Ley RE, van der Meulen MC, Goldring SR, Hernandez CJ. Alterations to the Gut Microbiome Impair Bone Strength and Tissue Material Properties. J Bone Miner Res. 2017; 32:1343–53. 10.1002/jbmr.3114 28244143 PMC5466506

[17] Wang W, Wang ZP, Huang CY, Chen YD, Yao WF, Shi BM. The Neuropeptide Vasoactive Intestinal Peptide Levels in Serum are Inversely Related to Disease Severity of Postmenopausal Osteoporosis: A Cross-Sectional Study. Genet Test Mol Biomarkers. 2019; 23:480–86. 10.1089/gtmb.2019.0041 31157986

[18] Tyagi AM, Yu M, Darby TM, Vaccaro C, Li JY, Owens JA, Hsu E, Adams J, Weitzmann MN, Jones RM, Pacifici R. The Microbial Metabolite Butyrate Stimulates Bone Formation via T Regulatory Cell-Mediated Regulation of WNT10B Expression. Immunity. 2018; 49:1116–1131.e7. 10.1016/j.immuni.2018.10.013 30446387 PMC6345170

[19] Stotzer PO, Johansson C, Mellström D, Lindstedt G, Kilander AF. Bone mineral density in patients with small intestinal bacterial overgrowth. Hepatogastroenterology. 2003; 50:1415–18. 14571751

[20] Sjögren K, Engdahl C, Henning P, Lerner UH, Tremaroli V, Lagerquist MK, Bäckhed F, Ohlsson C. The gut microbiota regulates bone mass in mice. J Bone Miner Res. 2012; 27:1357–67. 10.1002/jbmr.1588 22407806 PMC3415623

[21] Yan J, Herzog JW, Tsang K, Brennan CA, Bower MA, Garrett WS, Sartor BR, Aliprantis AO, Charles JF. Gut microbiota induce IGF-1 and promote bone formation and growth. Proc Natl Acad Sci USA. 2016; 113:E7554–63. 10.1073/pnas.1607235113 27821775 PMC5127374

[22] Wang J, Wang Y, Gao W, Wang B, Zhao H, Zeng Y, Ji Y, Hao D. Diversity analysis of gut microbiota in osteoporosis and osteopenia patients. PeerJ. 2017; 5:e3450. 10.7717/peerj.3450 28630804 PMC5474093

[23] Das M, Cronin O, Keohane DM, Cormac EM, Nugent H, Nugent M, Molloy C, O'Toole PW, Shanahan F, Molloy MG, Jeffery IB. Gut microbiota alterations associated with reduced bone mineral density in older adults. Rheumatology (Oxford). 2019; 58:2295–2304. 10.1093/rheumatology/kez302 31378815 PMC6880854

[24] Li C, Huang Q, Yang R, Dai Y, Zeng Y, Tao L, Li X, Zeng J, Wang Q. Gut microbiota composition and bone mineral loss-epidemiologic evidence from individuals in Wuhan, China. Osteoporos Int. 2019; 30:1003–13. 10.1007/s00198-019-04855-5 30666372

[25] Kalkan R, Tulay P. The Interactions between Bone Remodelling, Estrogen Hormone and EPH Family Genes. Crit Rev Eukaryot Gene Expr. 2018; 28:135–38. 10.1615/CritRevEukaryotGeneExpr.2018021275 30055540

[26] Kanis JA, Cooper C, Rizzoli R, Reginster JY, and Scientific Advisory Board of the European Society for Clinical and Economic Aspects of Osteoporosis and Osteoarthritis (ESCEO) and the Committees of Scientific Advisors and National Societies of the International Osteoporosis Foundation (IOF). Executive summary of European guidance for the diagnosis and management of osteoporosis in postmenopausal women. Aging Clin Exp Res. 2019; 31:15–17. 10.1007/s40520-018-1109-4 30612282

[27] Vasikaran S, Cooper C, Eastell R, Griesmacher A, Morris HA, Trenti T, Kanis JA. International Osteoporosis Foundation and International Federation of Clinical Chemistry and Laboratory Medicine position on bone marker standards in osteoporosis. Clin Chem Lab Med. 2011; 49:1271–74. 10.1515/CCLM.2011.602 21605012

[28] Edgar RC. UPARSE: highly accurate OTU sequences from microbial amplicon reads. Nat Methods. 2013; 10:996–98. 10.1038/nmeth.2604 23955772

[29] Harre U, Lang SC, Pfeifle R, Rombouts Y, Frühbeißer S, Amara K, Bang H, Lux A, Koeleman CA, Baum W, Dietel K, Gröhn F, Malmström V, et al. Glycosylation of immunoglobulin G determines osteoclast differentiation and bone loss. Nat Commun. 2015; 6:6651. 10.1038/ncomms7651 25825024 PMC4389255

[30] Park SG, Jeong SU, Lee JH, Ryu SH, Jeong HJ, Sim YJ, Kim DK, Kim GC. The Changes of CTX, DPD, Osteocalcin, and Bone Mineral Density During the Postmenopausal Period. Ann Rehabil Med. 2018; 42:441–48. 10.5535/arm.2018.42.3.441 29961742 PMC6058582

[31] Xu X, Jia X, Mo L, Liu C, Zheng L, Yuan Q, Zhou X. Intestinal microbiota: a potential target for the treatment of postmenopausal osteoporosis. Bone Res. 2017; 5:17046. 10.1038/boneres.2017.46 28983411 PMC5627629

[32] McCabe LR, Irwin R, Schaefer L, Britton RA. Probiotic use decreases intestinal inflammation and increases bone density in healthy male but not female mice. J Cell Physiol. 2013; 228:1793–98. 10.1002/jcp.24340 23389860 PMC4091780

[33] Takada T, Kurakawa T, Tsuji H, Nomoto K. Fusicatenibacter saccharivorans gen. nov., sp. nov., isolated from human faeces. Int J Syst Evol Microbiol. 2013; 63:3691–96. 10.1099/ijs.0.045823-0 23625266

[34] Duncan SH, Barcenilla A, Stewart CS, Pryde SE, Flint HJ. Acetate utilization and butyryl coenzyme A (CoA):acetate-CoA transferase in butyrate-producing bacteria from the human large intestine. Appl Environ Microbiol. 2002; 68:5186–90. 10.1128/aem.68.10.5186-5190.2002 12324374 PMC126392

[35] Ríos-Covián D, Ruas-Madiedo P, Margolles A, Gueimonde M, de Los Reyes-Gavilán CG, Salazar N. Intestinal Short Chain Fatty Acids and their Link with Diet and Human Health. Front Microbiol. 2016; 7:185. 10.3389/fmicb.2016.00185 26925050 PMC4756104

[36] Inoue R, Ohue-Kitano R, Tsukahara T, Tanaka M, Masuda S, Inoue T, Yamakage H, Kusakabe T, Hasegawa K, Shimatsu A, Satoh-Asahara N. Prediction of functional profiles of gut microbiota from 16S rRNA metagenomic data provides a more robust evaluation of gut dysbiosis occurring in Japanese type 2 diabetic patients. J Clin Biochem Nutr. 2017; 61:217–21. 10.3164/jcbn.17-44 29203964 PMC5703784

[37] Chen W, Liu F, Ling Z, Tong X, Xiang C. Human intestinal lumen and mucosa-associated microbiota in patients with colorectal cancer. PLoS One. 2012; 7:e39743. 10.1371/journal.pone.0039743 22761885 PMC3386193

[38] Qin N, Yang F, Li A, Prifti E, Chen Y, Shao L, Guo J, Le Chatelier E, Yao J, Wu L, Zhou J, Ni S, Liu L, et al. Alterations of the human gut microbiome in liver cirrhosis. Nature. 2014; 513:59–64. 10.1038/nature13568 25079328

[39] Tyler AD, Knox N, Kabakchiev B, Milgrom R, Kirsch R, Cohen Z, McLeod RS, Guttman DS, Krause DO, Silverberg MS. Characterization of the gut-associated microbiome in inflammatory pouch complications following ileal pouch-anal anastomosis. PLoS One. 2013; 8:e66934. 10.1371/journal.pone.0066934 24086242 PMC3782502

[40] D'Amelio P, Grimaldi A, Di Bella S, Brianza SZM, Cristofaro MA, Tamone C, Giribaldi G, Ulliers D, Pescarmona GP, Isaia G. Estrogen deficiency increases osteoclastogenesis up-regulating T cells activity: a key mechanism in osteoporosis. Bone. 2008; 43:92–100. 10.1016/j.bone.2008.02.017 18407820

[41] Hsu E, Pacifici R. From Osteoimmunology to Osteomicrobiology: How the Microbiota and the Immune System Regulate Bone. Calcif Tissue Int. 2018; 102:512–21. 10.1007/s00223-017-0321-0 29018933 PMC5893441

[42] Mafra D, Lobo JC, Barros AF, Koppe L, Vaziri ND, Fouque D. Role of altered intestinal microbiota in systemic inflammation and cardiovascular disease in chronic kidney disease. Future Microbiol. 2014; 9:399–410. 10.2217/fmb.13.165 24762311

[43] Rizzatti G, Lopetuso LR, Gibiino G, Binda C, Gasbarrini A. Proteobacteria: A Common Factor in Human Diseases. Biomed Res Int. 2017; 2017:9351507. 10.1155/2017/9351507 29230419 PMC5688358

[44] Shin NR, Whon TW, Bae JW. Proteobacteria: microbial signature of dysbiosis in gut microbiota. Trends Biotechnol. 2015; 33:496–503. 10.1016/j.tibtech.2015.06.011 26210164

[45] Lieben L, Masuyama R, Torrekens S, Van Looveren R, Schrooten J, Baatsen P, Lafage-Proust MH, Dresselaers T, Feng JQ, Bonewald LF, Meyer MB, Pike JW, Bouillon R, Carmeliet G. Normocalcemia is maintained in mice under conditions of calcium malabsorption by vitamin D-induced inhibition of bone mineralization. J Clin Invest. 2012; 122:1803–15. 10.1172/JCI45890 22523068 PMC3336970

[46] Rosen HN, Rosen CJ, Schmader KE, Mulder JE. Calcium and vitamin D supplementation in osteoporosis. 2019 https://www.uptodate.com/contents/calcium-and-vitamin-d-supplementation-in-osteoporosis

[47] Pizzino G, Irrera N, Galfo F, Oteri G, Atteritano M, Pallio G, Mannino F, D’Amore A, Pellegrino E, Aliquò F, Anastasi GP, Cutroneo G, Squadrito F, et al. Adenosine Receptor Stimulation Improves Glucocorticoid-Induced Osteoporosis in a Rat Model. Front Pharmacol. 2017; 8:558. 10.3389/fphar.2017.00558 28928654 PMC5591884

[48] Mediero A, Cronstein BN. Adenosine and bone metabolism. Trends Endocrinol Metab. 2013; 24:290–300. 10.1016/j.tem.2013.02.001 23499155 PMC3669669

[49] Carroll SH, Wigner NA, Kulkarni N, Johnston-Cox H, Gerstenfeld LC, Ravid K. A2B adenosine receptor promotes mesenchymal stem cell differentiation to osteoblasts and bone formation in vivo. J Biol Chem. 2012; 287:15718–27. 10.1074/jbc.M112.344994 22403399 PMC3346096

[50] Shih YV, Liu M, Kwon SK, Iida M, Gong Y, Sangaj N, Varghese S. Dysregulation of ectonucleotidase-mediated extracellular adenosine during postmenopausal bone loss. Sci Adv. 2019; 5:eaax1387. 10.1126/sciadv.aax1387 31457100 PMC6703860

[51] Zhang DW, Wang ZL, Qi W, Lei W, Zhao GY. Cordycepin (3′-deoxyadenosine) down-regulates the proinflammatory cytokines in inflammation-induced osteoporosis model. Inflammation. 2014; 37:1044–49. 10.1007/s10753-014-9827-z 24493324

[52] Visalakshi RM, Suresh V. Effect of Aminoacids Arginine and Lysine on Osteoblastic Activity. J Pharm Sci Res. 2016; 8:1021 https://www.jpsr.pharmainfo.in/Documents/Volumes/vol8Issue09/jpsr08091613.pdf

[53] Wang HY, Hu P, Jiang J. Pharmacokinetics and safety of calcium L-threonate in healthy volunteers after single and multiple oral administrations. Acta Pharmacol Sin. 2011; 32:1555–60. 10.1038/aps.2011.138 21986570 PMC4010217

[54] Wan Y, Wang F, Yuan J, Li J, Jiang D, Zhang J, Li H, Wang R, Tang J, Huang T, Zheng J, Sinclair AJ, Mann J, Li D. Effects of dietary fat on gut microbiota and faecal metabolites, and their relationship with cardiometabolic risk factors: a 6-month randomised controlled-feeding trial. Gut. 2019; 68:1417–29. 10.1136/gutjnl-2018-317609 30782617

[55] Barcik W, Wawrzyniak M, Akdis CA, O'Mahony L. Immune regulation by histamine and histamine-secreting bacteria. Curr Opin Immunol. 2017; 48:108–13. 10.1016/j.coi.2017.08.011 28923468

[56] Koohdar VA, Razavilar V, Motalebi AA, Mosakhani F, Valinassab T. Isolation and Identification of Histamine-forming bacteria in frozen Skipjack tuna (Katsuwonus pelamis). Iranian Journal of Fisheries Sciences. 2011; 10:678–88. https://pdfs.semanticscholar.org/38a4/ce9d9be670005db092b58b7feed0ceb7b679.pdf

[57] Zhang J, Zhu X, Xu R, Gao Q, Wang D, Zhang Y. Isolation and identification of histamine-producing Enterobacteriaceae from Qu fermentation starter for Chinese rice wine brewing. Int J Food Microbiol. 2018; 281:1–9. 10.1016/j.ijfoodmicro.2018.05.014 29800825

[58] Kinjo M, Setoguchi S, Solomon DH. Antihistamine therapy and bone mineral density: analysis in a population-based US sample. Am J Med. 2008; 121:1085–91. 10.1016/j.amjmed.2008.06.036 19028205 PMC2943241

[59] Parveen S, Saravanan DB, Saluja R, Elden BT. IL-33 mediated amplification of allergic response in human mast cells. J Recept Signal Transduct Res. 2019; 39:359–67. 10.1080/10799893.2019.1690515 31755331

[60] Hodzic Z, Schill EM, Bolock AM, Good M. IL-33 and the intestine: the good, the bad, and the inflammatory. Cytokine. 2017; 100:1–10. 10.1016/j.cyto.2017.06.017 28687373 PMC5650929

[61] Ginaldi L, De Martinis M, Saitta S, Sirufo MM, Mannucci C, Casciaro M, Ciccarelli F, Gangemi S. Interleukin-33 serum levels in postmenopausal women with osteoporosis. Sci Rep. 2019; 9:3786. 10.1038/s41598-019-40212-6 30846811 PMC6405990

[62] Kharroubi A, Saba E, Ghannam I, Darwish H. Evaluation of the validity of osteoporosis and fracture risk assessment tools (IOF One Minute Test, SCORE, and FRAX) in postmenopausal Palestinian women. Arch Osteoporos. 2017; 12:6. 10.1007/s11657-016-0298-8 28013446

[63] Compston JE, McClung MR, Leslie WD. Osteoporosis. Lancet. 2019; 393:364–76. 10.1016/S0140-6736(18)32112-3 30696576

[64] Henriksen K, Christiansen C, Karsdal MA. Role of biochemical markers in the management of osteoporosis. Climacteric. 2015; 18:10–8. 10.3109/13697137.2015.1101256 26507704

[65] Chambers MC, Maclean B, Burke R, Amodei D, Ruderman DL, Neumann S, Gatto L, Fischer B, Pratt B, Egertson J, Hoff K, Kessner D, Tasman N, et al. A cross-platform toolkit for mass spectrometry and proteomics. Nat Biotechnol. 2012; 30:918–20. 10.1038/nbt.2377 23051804 PMC3471674

[66] Kuhl C, Tautenhahn R, Böttcher C, Larson TR, Neumann S. CAMERA: an integrated strategy for compound spectra extraction and annotation of liquid chromatography/mass spectrometry data sets. Anal Chem. 2012; 84:283–89. 10.1021/ac202450g 22111785 PMC3658281

[67] Martin M. Cutadapt removes adapter sequences from high-throughput sequencing reads. EMBnet J. 2011; 17:10–2. 10.14806/ej.17.1.200

[68] Quast C, Pruesse E, Yilmaz P, Gerken J, Schweer T, Yarza P, Peplies J, Glöckner FO. The SILVA ribosomal RNA gene database project: improved data processing and web-based tools. Nucleic Acids Res. 2013; 41:D590–6. 10.1093/nar/gks1219 23193283 PMC3531112

[69] Callahan BJ, McMurdie PJ, Rosen MJ, Han AW, Johnson AJ, Holmes SP. DADA2: High-resolution sample inference from Illumina amplicon data. Nat Methods. 2016; 13:581–3. 10.1038/nmeth.3869 27214047 PMC4927377

[70] McMurdie PJ, Holmes S. phyloseq: an R package for reproducible interactive analysis and graphics of microbiome census data. PLoS One. 2013; 8:e61217. 10.1371/journal.pone.0061217 23630581 PMC3632530

[71] Oksanen J, Blanchet FG, Friendly M, Kindt R, Legendre P, McGlinn D, Minchin PR, O’Hara RB, Simpson GL, Solymos P, Stevens MHH, Szoecs E, Wagner H. vegan: Community Ecology Package. 2019 https://cran.r-project.org/web/packages/vegan/index.html

[72] Pedersen TL. ggraph: An Implementation of Grammar of Graphics for Graphs and Networks. 2019 https://cran.r-project.org/web/packages/ggraph/index.html

[73] Wickham H. Data Analysis. In: ggplot2. Use R!. Springer, Cham 2016 p.189–201. 10.1007/978-3-319-24277-4_9

