# Supplementary material for: Gut microbiota and metabolite alterations associated with reduced bone mineral density or bone metabolic indexes in postmenopausal osteoporosis
Source: Aging (Albany NY). 2020 May 11;12(9):8583–604. doi: 10.18632/aging.103168 (PMC7244073; doi:10.18632/aging.103168)
Supplement: Supplementary Figures [file aging-12-103168-s003..pdf]

SUPPLEMENTARY FIGURES

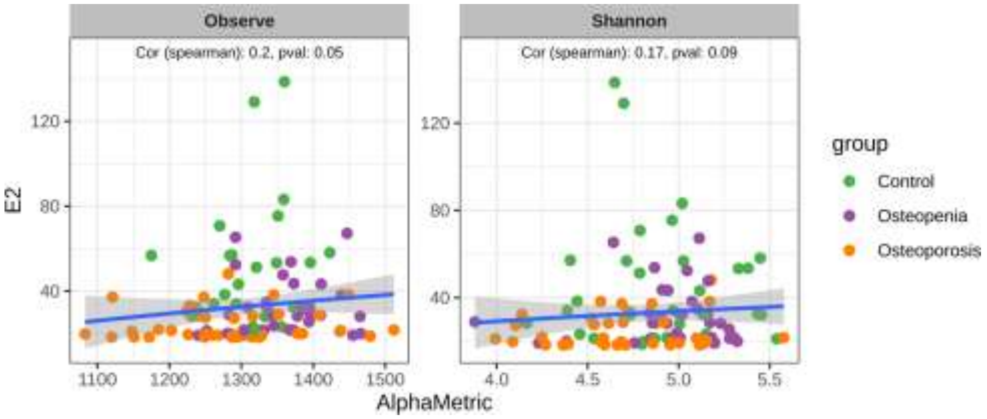

Supplementary Figure 1. Correlation between bacterial diversity and E_2. The x-axis shows the diversity values, and the y-axis shows the E2. The correlation is calculated with spearman method.

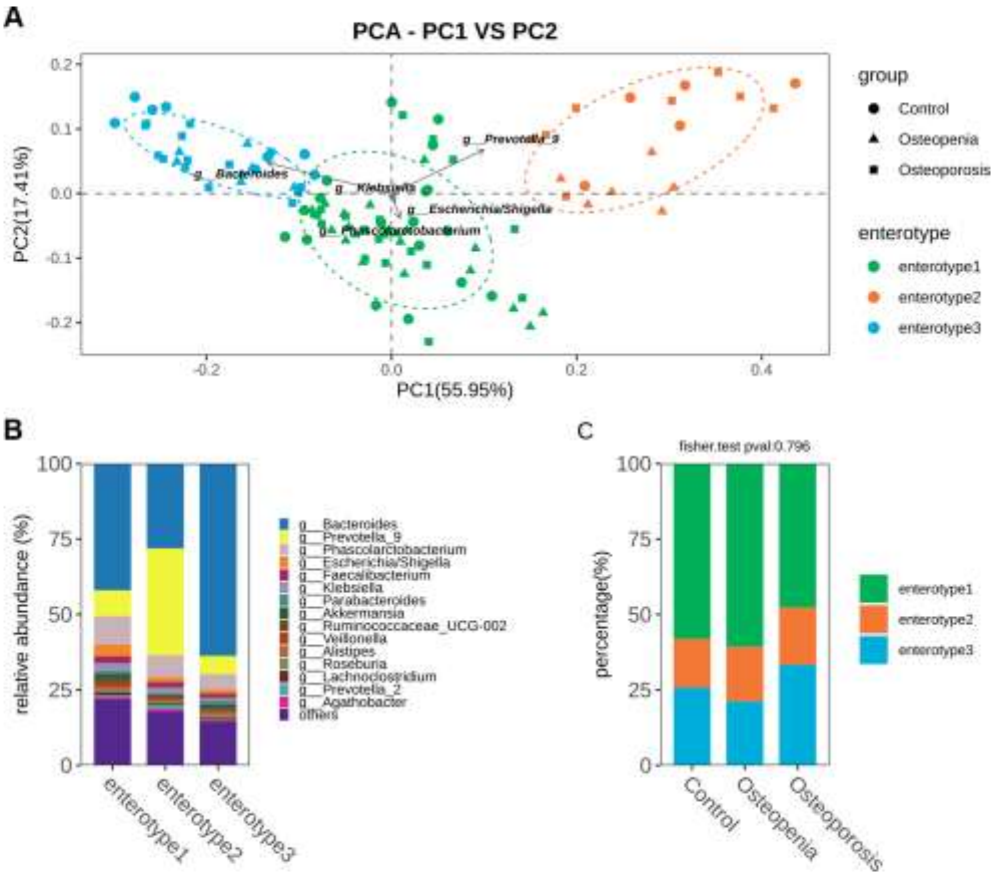

Supplementary Figure 2. No shift of gut enterotypes in postmenopausal osteoporosis and osteopenia. (**A**) Total samples are clustered into three types of enterotypes, the major contributors in the three enterotypes are *Klebsiella* (*Phascolarctobacterium*, *Escherichia/Shigella*), *Prevotella_9,* and *Bacterodies,* respectively. (**B**) Relative abundance of the top genera in the three enterotypes. (**C**) Proportions of enterotypes in each group. No statistically significant differences were observed among the groups.

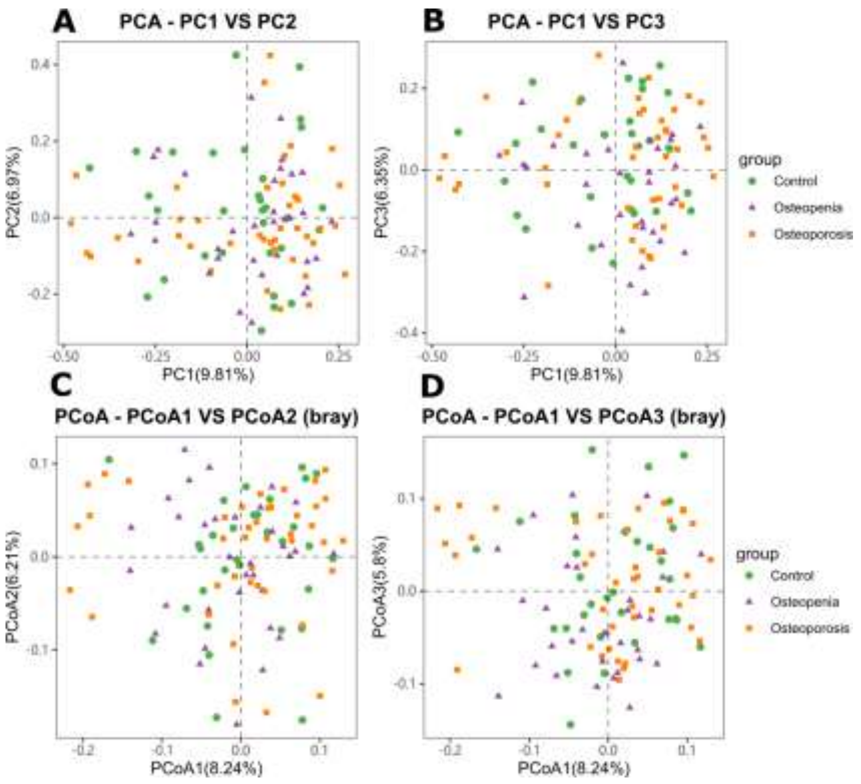

Supplementary Figure 3. PCA (Principal components analysis) and PCoA (Principal coordinate analysis) of bacterial β-diversity in the three groups. (**A**) Clustering of the first two principal components. (**B**) Clustering of the first principal components and third principal components. (**C**) Clustering of the first two principal coordinates. (**D**) Clustering of first principal coordinates and third principal coordinates.

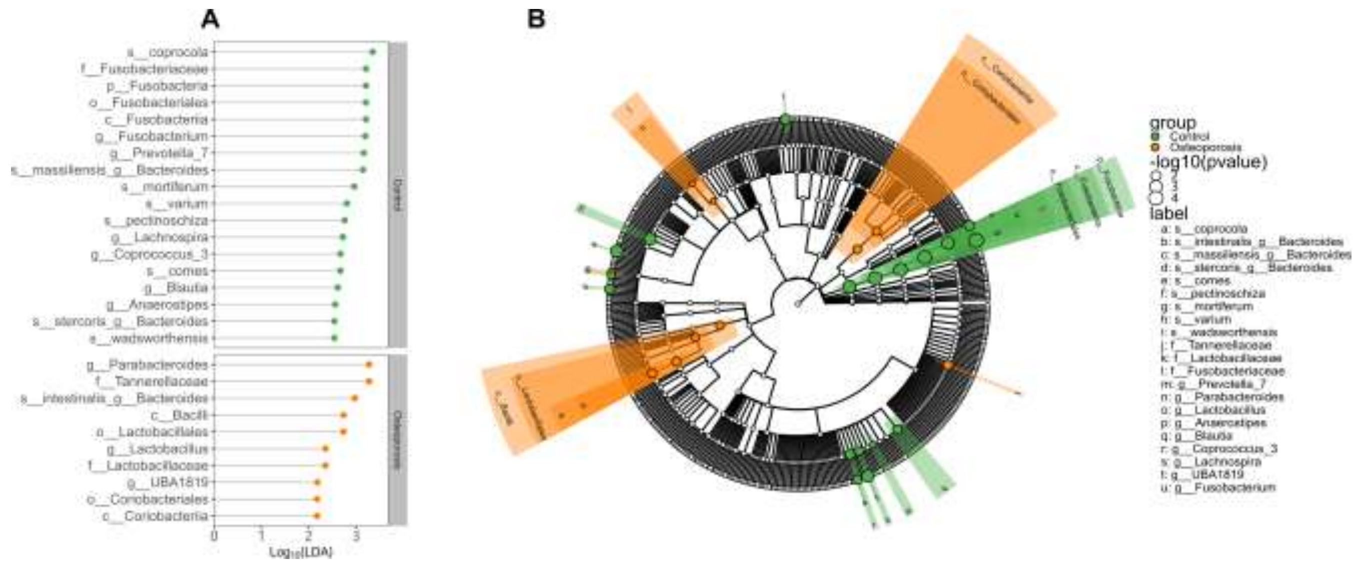

Supplementary Figure 4. Discriminative taxa between postmenopausal osteoporosis and control groups. (**A**) The point plot of LDA (Linear discriminant analysis) shows the features detected as statistically and biologically differential taxa between the different communities. (**B**) The taxonomic representation of statistically and biologically differences between postmenopausal osteoporosis and control. The color of discriminative taxa represents the taxa is more abundant in the corresponding group (control in green, postmenopausal osteoporosis in orange). The size of point shows the negative logarithms (base 10) of p-value. The bigger size of point shows more significant (lower p-value).

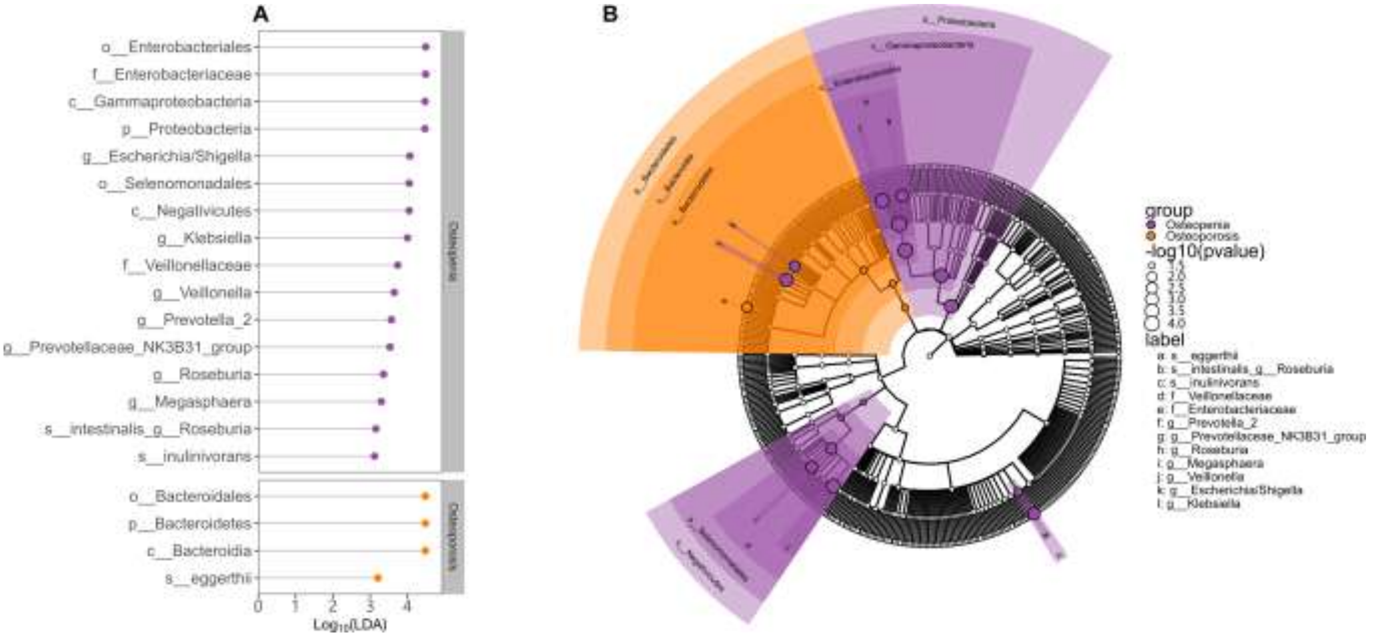

Supplementary Figure 5. Discriminative taxa between postmenopausal osteopenia and postmenopausal osteoporosis groups. (**A**) The point plot of LDA (Linear discriminant analysis) shows the features detected as statistically and biologically differential taxa between the different communities. (**B**) The taxonomic representation of statistically and biologically differences between postmenopausal osteopenia and postmenopausal osteoporosis. The colors of discriminative taxa represent the taxa is more abundant in the corresponding group (postmenopausal osteopenia in purple, postmenopausal osteoporosis in orange), the size of point shows the negative logarithms (base 10) of p-value. The bigger size of point shows more significant (lower p-value).

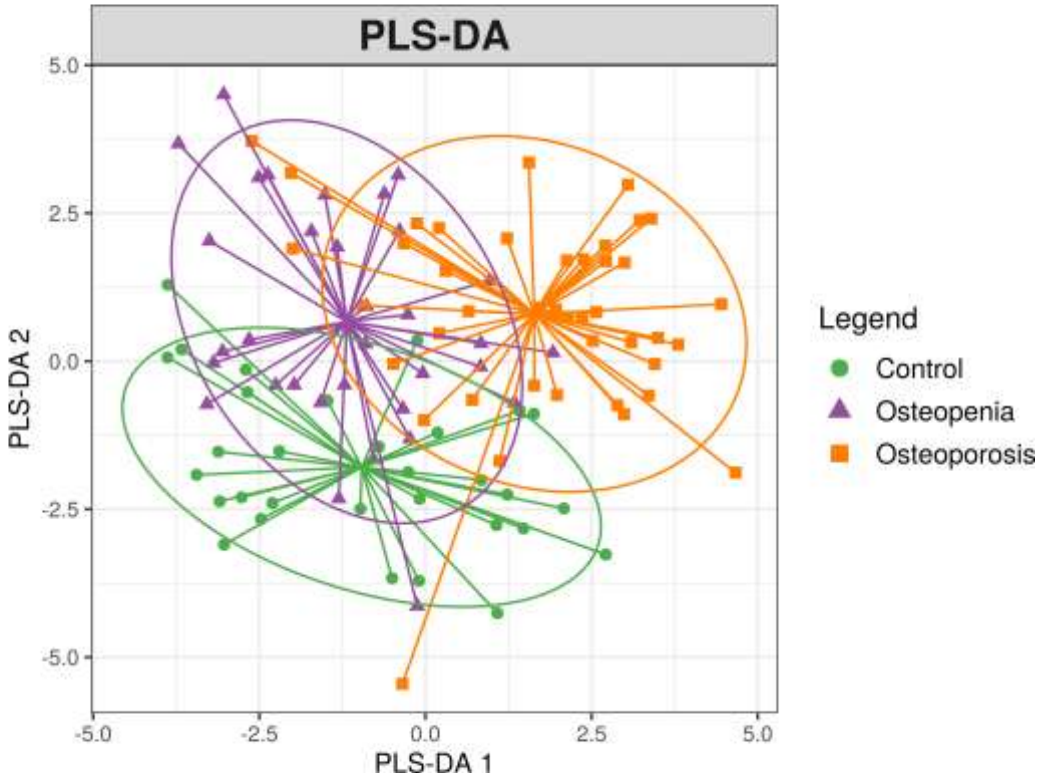

Supplementary Figure 6. PLS-DA score plots comparing the fecal metabolites in the three groups.

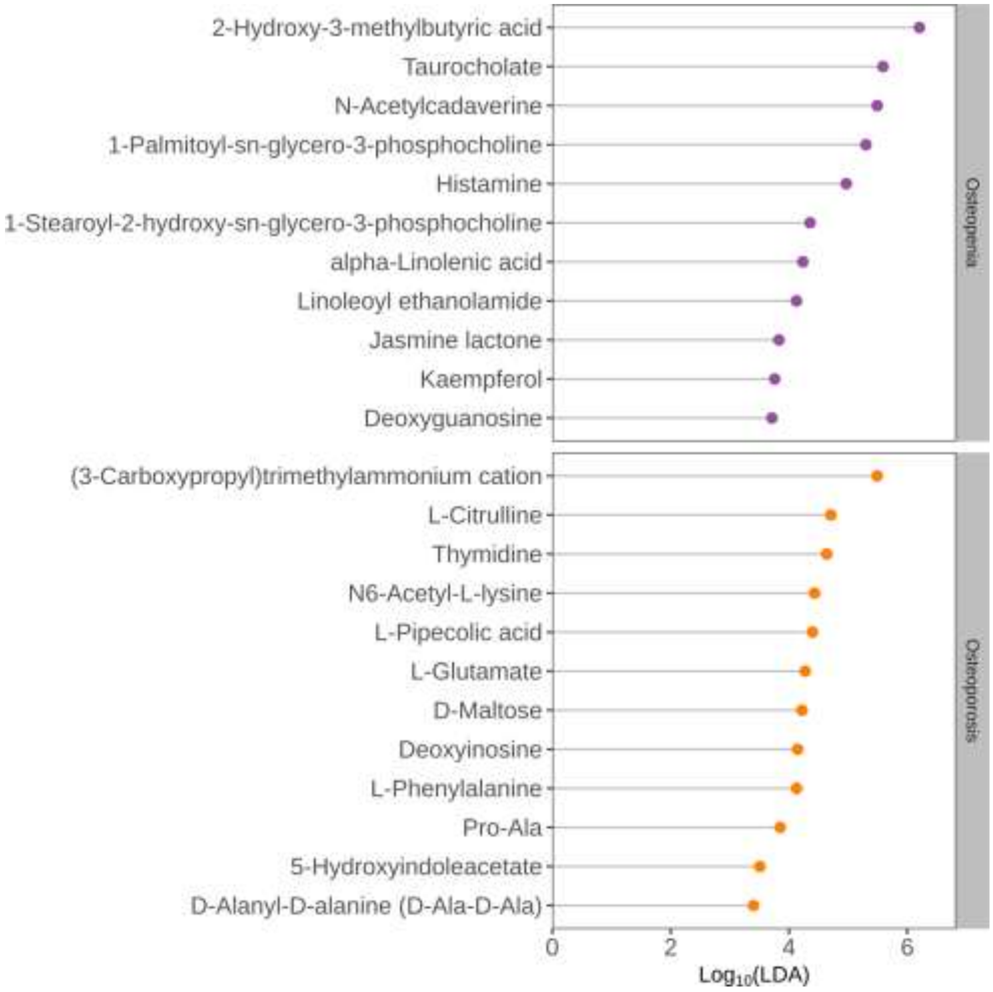

Supplementary Figure 7. Discriminative fecal metabolites between postmenopausal osteopenia (purple) and postmenopausal osteoporosis (orange). The x-axis shows the logarithms (base 10) of LDA (Linear discriminant analysis). The y-axis shows the discriminiative fecal metabolites.
