# Supplementary material for: Gut microbiota and metabolite alterations associated with reduced bone mineral density or bone metabolic indexes in postmenopausal osteoporosis
Source: Aging (Albany NY). 2020 May 11;12(9):8583–604. doi: 10.18632/aging.103168 (PMC7244073; doi:10.18632/aging.103168)
Supplement: Supplementary Tables 1-3 [file aging-12-103168-s002..pdf]

SUPPLEMENTARY TABLES

Supplementary Table 1. Characteristics of the participants involved in this study.

	Meta-data	Normal BMD (n=31)	Osteopenia (n=33)	Osteoporosis (n=42)	p-value	Normal BMD - Osteopenia	Normal BMD-Osteoporosis	Osteopenia-Osteoporosis
Basic data	Age(years)	57.35±3.98	57.42±5.06	59.69±5.51	0.137	1	0.112	0.19
	weight (kg)	60.71±6.6	58.79±7.5	57.29±5.85	0.207	0.248	0.031	0.332
	BMI (kg/m2)	24.28±2.79	24.21±3.05	23.8±2.17	0.737	1	0.818	0.89
Bone density test	LS Z-score	1.20±1.06	-0.49±0.62	-1.65±0.74	<0.001	<0.001	<0.001	<0.001
	LS T-score	0.06±0.93	-1.69±0.56	-3.14±0.62	<0.001	<0.001	<0.001	<0.001
	LS BMD(g/cm2)	1.19±0.11	0.98±0.07	0.80±0.07	<0.001	<0.001	<0.001	<0.001
	FN Z-score	1.10±0.74	-0.12±0.62	-0.83±0.76	<0.001	<0.001	<0.001	<0.001
	FN T-score	-0.01±0.69	-1.16±0.77	-2.21±0.78	<0.001	<0.001	<0.001	<0.001
	FN BMD(g/cm2)	0.98±0.08	0.83±0.08	0.72±0.10	<0.001	<0.001	<0.001	<0.001
	Total hip Z-score	0.98±0.77	-0.10±0.66	-0.92±0.87	<0.001	<0.001	<0.001	<0.001
	Total hip T-score	0.13±0.72	-1.00±0.67	-2.00±0.92	<0.001	<0.001	<0.001	<0.001
	Total hip BMD(g/cm2)	1.02±0.09	0.88±0.08	0.76±0.11	<0.001	<0.001	<0.001	<0.001
Blood indices	E2(pmol/L)	45.85±29.35	31.94±13.02	24.42±7.47	<0.001	0.058	0.001	0.014
	25(OH)VD3(nmol/L)	50.86±17.7	44.35±15.38	56.28±20.46	0.126	0.155	0.212	0.006
	OC(ng/ml)	19.96±7.45	26.29±10.03	24.24±13.25	0.031	0.017	0.231	0.831
	CTX-1(ng/ml)	0.38±0.18	0.56±0.24	0.48±0.33	0.021	0.004	0.253	0.557
	P1NP(ng/ml)	54.92±21.35	70.61±26.3	64.91±43.46	0.024	0.032	0.486	0.861
	PTH(pg/ml)	45.4±21.59	45.02±16.42	47.65±26.08	0.287	0.946	0.67	0.612
Living habits	Smoking Status (Yes/No)	1/30	2/31	2/40	0.863			
	Drinking Status (Yes/No)	2/29	1/32	3/39	0.703			
Chronic diseases	Diabetes Type II (Yes/No)	4/27	5/28	6/36	0.967			
	Hypertension (Yes/No)	5/26	6/27	8/34	0.949			
	Hyperlipemia (Yes/No)	5/26	4/29	3/39	0.475			
	Chronic gastritis (Yes/No)	2/29	1/32	1/41	0.664			
	Osteoarthritis (Yes/No)	2/29	2/31	2/40	0.946			
Medications	Number of medications	1±0.97	0.79±0.99	0.88±0.89	0.669			
	Metformin (Yes/No)	2/29	2/31	2/40	0.946			
	Dipeptidyl peptidase-4 (DDP-4) inhibitors (Yes/No)	1/30	2/31	3/39	0.75			
	Insulin(Yes/No)	1/30	1/32	2/40	0.912			
	β-blockers (Yes/No)	1/30	2/31	1/41	0.709			

ARBs ACEi (Yes/No)	5/26	6/27	8/34	0.949
Cholesterol medications (Yes/No)	5/26	4/29	3/39	0.475
PPIs (Yes/No)	2/29	1/32	1/41	0.664
NSAIDs (Yes/No)	2/29	2/31	2/40	0.946
Nutritional Supplements (Yes/No)	3/28	3/30	5/37	0.914
Calcium supplements (Yes/No)	3/28	2/31	4/38	0.824
Vitamins (Yes/No)	3/28	1/32	5/37	0.322

Supplementary Table 2. The results of PERMUNATION ANOVA analysis based on the OTU profilers in the three groups.

Factors	Df	SumsOfSqs	MeanSqs	F.Model	R2	pvalue	compare	FDR
group	2	0.65122	0.32561	1.929175	0.036213	4.00E-04	three group	0.0024
E2	1	0.183476	0.183476	1.087061	0.010203	0.3152	three group	0.556629
group:E2	2	0.269903	0.134952	0.799561	0.015009	0.8892	three group	0.9568
group	1	0.384969	0.384969	2.479287	0.038481	3.00E-04	Control-VS-Osteopenia	0.0024
E2	1	0.184224	0.184224	1.186442	0.018415	0.2101	Control-VS-Osteopenia	0.50424
group:E2	1	0.118545	0.118545	0.763453	0.011849	0.8325	Control-VS-Osteopenia	0.9568
group	1	0.218132	0.218132	1.266553	0.017541	0.139	Control-VS-Osteoporosis	0.417
E2	1	0.186088	0.186088	1.080493	0.014964	0.3247	Control-VS-Osteoporosis	0.556629
group:E2	1	0.148086	0.148086	0.859841	0.011908	0.6715	Control-VS-Osteoporosis	0.914267
group	1	0.379569	0.379569	2.146261	0.028743	0.0012	Osteopenia-VS-Osteoporosis	0.0048
E2	1	0.116269	0.116269	0.657438	0.008804	0.9568	Osteopenia-VS-Osteoporosis	0.9568
group:E2	1	0.153548	0.153548	0.868233	0.011627	0.6857	Osteopenia-VS-Osteoporosis	0.914267

Supplementary Table 3. The results of orthogonal projection to latent structure-discriminant analysis and PERMUNATION ANOVA analysis based on fecal metobolites profilers in the three groups.

R2X(cum)	R2Y(cum)	Q2(cum)	RMSEE	pre	ort	pR2Y	pQ2	fdr	comparison
0.19	0.28	0.0072	0.403	2	0	0.1	0.066666667	0.088889	three groups
0.197	0.688	0.319	0.286	2	0	0.14	0.006666667	0.013333	Control_vs_Osteopenia
0.161	0.697	0.252	0.278	2	0	0.026667	0.006666667	0.013333	Control_vs_Osteoporosis
0.172	0.593	0.0524	0.323	2	0	0.28	0.136666667	0.136667	Osteopenia_vs_Osteoporosis

Factors	Df	SumsOfSqs	MeanSqs	F.Model	R2	pvalue	compare	FDR
group	2	0.40969119	0.204846	1.559638	0.029513	0.0395	three group	0.0954
group	1	0.1775052	0.177505	1.366989	0.021595	0.16	Control-VS-Osteopenia	0.16
group	1	0.22537407	0.225374	1.737694	0.024098	0.0477	Control-VS-Osteoporosis	0.0954
group	1	0.20819501	0.208195	1.551384	0.020854	0.0854	Osteopenia-VS-Osteoporosis	0.113867
